# Sex Differences in Time to Treat and Outcomes for Gliomas

**DOI:** 10.3389/fonc.2021.630597

**Published:** 2021-02-19

**Authors:** Nickolas Stabellini, Halle Krebs, Nirav Patil, Kristin Waite, Jill S. Barnholtz-Sloan

**Affiliations:** ^1^ Department of Population Health and Quantitative Sciences, Case Western Reserve University School of Medicine, Cleveland, OH, United States; ^2^ Faculdade Israelita de Ciências da Saúde Albert Einstein (FICSAE), Hospital Israelita Albert Einstein, São Paulo, Brazil; ^3^ The Ohio State University, Department of Biology, Columbus, OH, United States; ^4^ Central Brain Tumor Registry of the United States (CBTRUS), Hinsdale, IL, United States; ^5^ Research and Education Institute, University Health System, Cleveland, OH, United States; ^6^ Cleveland Center for Health Outcomes Research (CCHOR), Cleveland, OH, United States; ^7^ Case Comprehensive Cancer Center, Cleveland, OH, United States; ^8^ Research Health Analytics and Informatics, University Hospitals Health System, Cleveland, OH, United States

**Keywords:** gliomas, glioblastoma, outcomes, sex differences, National Cancer Database, treatment

## Abstract

**Background:**

Gliomas are the most common type of primary malignant brain tumor in adults, representing one third of all primary and central nervous system (CNS) tumors and 80% of malignant tumors diagnosed in the Western world. Epidemiological data indicate that the overall incidence and mortality of cancer is higher in males, while females have a better prognosis. The goal of this study is to determine whether there are sex differences in the time to treat and clinical outcomes in patients with glioma

**Methods:**

Glioblastoma (GB) and Lower Grade Glioma (LGG) patients were defined per the Central Brain Tumor Registry of the United States (CBTRUS) from the National Cancer Database (NCDB) for diagnosis years 2004 to 2016. Associations between sex and time to treatment variables as well as associations between sex and multiple clinical outcomes were assessed using univariable and multivariable models.

**Results:**

A total of 176,100 patients were used for analysis (124,502 GBM and 51,598 LGG). Males had a statistically significant association with >7 days to surgery (OR = 1.09, CI 1.05–1.13, p < 0.001) but this association was not observed in the multivariable model (OR = 1.05, CI 0.96–1.16, p = 0.25). After adjustment for key variables including time to treat variables, males with GB and LGG had a higher risk of death (HR = 1.11, CI 1.09–1.13, p < 0.001, HR = 1.09, CI 1.03–1.15, p < 0.001; respectfully). Sex differences in 90-day mortality for GBM were not found after adjustment (OR for males = 0.99, CI 0.91–1.08, p = 0.93). For LGG, both the univariable and multivariable logistic regression models showed no sex differences in 90-day mortality (OR for males = 1.03, CI 0.94–1.12, p = 0.45; multivariable OR for males = 0.81, CI 0.62–1.06, p = 0.13).

**Conclusions:**

Based on NCDB data, there were no statistically significant differences in time to treatment between males and females, however males had a higher proportion of GB and LGG as well as a higher risk of death compared to females.

## Introduction

Gliomas are the most common type of primary malignant brain tumor in adults (1). These tumors represent one third of all primary and central nervous system (CNS) tumors and 80% of malignant tumors diagnosed in the Western world (2). Gliomas, per the World Health Organization (WHO) International Classification of Disease–Oncology (ICD-O) guidelines are classified based on histological and molecular criteria (3) and assigned grades I to IV.

Glioblastoma (GB), WHO grade IV, is the most common, aggressive, and deadly type of glioma, accounting for 48.3% of all primary malignant CNS tumors and 57.3% of all gliomas in the Western world (1, 4). With a median survival rate of 9 to 15 months and a 5-year survival rate of only 6.8%, GB has a poor prognosis (1, 5). The Stupp protocol which consists of surgical resection followed by concurrent radiotherapy and chemotherapy plus additional chemotherapy is the standard treatment for GB patients (6, 7). Lower grade gliomas, LGGs, are Grade II and III gliomas and represent approximately one third of all Gliomas (3). LGGs are more common in younger individuals, following an indolent course and have a good prognosis (8), with a 5-year survival rate reaching values of 98% (3, 8). Possible treatments involve observation, surgery, radiation, chemotherapy, or combination therapy (8).

When looking at cancer as a whole, epidemiological data indicate higher incidence rates (greater than 20%) and mortality rates (greater than 40%) in males (9), while females have a better prognosis cancer diagnosis (10). These sex-differences in incidence and survival also exist for brain tumors. GB is more common in males (age-adjusted incidence rate = 3,99/100.000) than females (age-adjusted incidence rate = 2,52/100.000) (11–13). Females with GB have a better median survival compared to males, even after adjustment for treatment patterns (13). The same is also seen in LGGs, where there is a higher incidence rate in males (14) and inconsistent reports of better survival in females (4, 15).

Despite reports of females with gliomas having better survival, the influencing factors for better survival are not fully known. We hypothesize that sex differences exist in the time to treatment for both GB and LGG which could be associated with differences in clinical outcomes for these tumors. Therefore, the primary objective of this work is to use the National Cancer Database (NCDB) to assess sex differences in time to treatment and analyze effects of these differences on survival for GB and LGG.

## Methods

Data were obtained from the NCDB for diagnosis years 2004 to 2016, a clinical oncology database sourced from hospital registry data that are collected in more than 1,500 Commission on Cancer (CoC) accredited facilities. NCBD data represents >70% of new cancer diagnoses in the United States (16). GB and LGG patients were defined per the Central Brain Tumor Registry of the United States (CBTRUS) (1), with International Classification of Disease–Oncology (version 3) histological codes 9440/3, 9441/3, and 9442/3 for GB and 9400/3, 9410/3, 9411/3, 9420/3, 9401/3, 9450/3, 9451/3, 9460/3, and 9382/3 for LGG. For the patients analyzed, the general characteristics were age at diagnosis, race, insurance, median income, location of residence, Charlson/Deyo Score, year of diagnosis, facility type, facility location, and distance from facility in miles. It should be noted that the facility type is only collected on patients older than 40 years old. As a result unknown facility type was considered as a separate category to include these patients in multivariable model. The time to treatment characteristics assessed were days from diagnosis to treatment, days from diagnosis to surgery, days until discharge, unplanned readmission after 30 days, days from diagnosis to radiation, days of radiation, and days from diagnosis to chemotherapy. Patients with missing time to treatment characteristics or missing treatment information were excluded from this analysis. Treatment variables assessed were surgery, radiation, and chemotherapy. A standard of care treatment variable was created for GBs only (Radiation + Chemotherapy + Surgery) (6). Clinical outcomes assessed were overall survival, 30-day mortality and 90-day mortality.

Patient level characteristics were compared by sex within histology group (GB or LGG) using the Chi-Square test for categorical variables and Mann-Whitney for continuous variables. When analyzing time for treatment variables, only patients who received the respective treatment were selected, excluding missing values. P<0.05 was considered significant.

Association between sex and overall days to treatment, days to surgery, days to discharge, days to radiation, and days to chemotherapy were assessed by Logistic Regression Models. Only patients who had received treatment with complete data for all variables of interest were included in the analysis. Univariable analysis was performed and variables with p < 0.20 were selected for the multivariable.

The influence of sex on multiple clinical outcomes (overall survival and 90-day mortality) was assessed using univariable and multivariable models. Overall survival by sex and histology was first assessed using Kaplan Meier analysis generating median survival by sex with 95% confidence intervals (95% CI) and log rank tests by sex. Cox proportional hazards regression models were used to assess univariable and multivariable models of overall survival by sex within each histology group (GB or LGG).

The influence of sex on 90-day mortality was assessed using univariable and multivariable logistic regression within each histology group (GB or LGG). Between the variables sex, age, insurance status, facility type (academic or non-academic cancer program), location of residence (urban or rural), facility region (East North Central, East South Central, Middle Atlantic, Mountain, New England, Pacific, South Atlantic, West North Central and West South Central), Charlson/Deyo Score, distance from facility, time to treat, time to surgery, time to radiation, and time to chemo only those with p < 0.20 were selected for all final multivariable models. Colinearity was assessed by the variance inflation factor (VIF). Due to low number of 30-day mortality, resulting in low sample size (5% of all population for GB and 2% for LGG), the influence of sex on this variable was not assessed using univariable and multivariable modeling.

All analyses were performed using R 3.6.3 software.

## Results

Using data from NCDB for the study years of 2004 to 2016, we analyzed a total of 176,100 patients (124,502 GB and 51,598 LGG) (**Table 1**). For GB patients 57.5% were male and 42.5% were female. Differences between males and females with GB existed for age (p < 0.001), race (p < 0.001), insurance status (p < 0.001), median income (p = 0.001), Charlson/Deyo Score (p < 0.001), facility type (p < 0.001), facility region (p = 0.006), and distance from facility (p < 0.001). There were no significant differences for location of residence (p = 0.25) and year of diagnosis (p = 0.37) for patients with GB. For LGG patients 55.6% were males and 44.4% females. Differences between males and females with LGG exist for age (p < 0.001), race (p = 0.003), insurance status (p < 0.001), Charlson/Deyo score (p < 0.001), and distance from facility (p < 0.001). There were no significant differences for median income (p = 0.85), location of residence (p = 0.51), years of diagnosis (p = 0.62), facility type (p = 0.15), and facility region (p = 0.40) for LGG patients.

**Table 1:** Patient characteristics by sex for Glioblastoma (GBM) and Lower Grade Gliomas (LGG), National Cancer Database (NCDB), 2004–2016.

	Glioblastoma (n = 124 502)			Lower Grade Glioma (n = 51 598)		
	Male (57.5%)	Female (42.5%)	P value	Male (55.6%)	Female (44.4%)	P value
**Age—n (%)**						
<40 y	3 847 (5.4%)	2 501 (4.7%)	<0.001^a^	9 745 (34%)	7 527 (32.8%)	<0.001^a^
40–64 y	34 447 (48.2%)	23 145 (43.7%)		13 099 (45.7%)	10 256 (44.7%)	
>= 65 y	33 238 (46.5%)	27 323 (51.6%)		5 820 (20.3%)	5 151 (22.5%)	
**Race—n (%)**						
White	64 868 (90.7%)	47 867 (90.4%)	<0.001^a^	25 469 (88.9%)	20 226 (88.2%)	0.003^a^
Black	3 823 (5.3%)	3 092 (5.8%)		1 670 (5.8%)	1 499 (6.5%)	
Other	2 842 (4%)	2 010 (3.8%)		1 525 (5.3%)	1 209 (5.3%)	
**Insurance—n (%)**						
Yes	67 124 (93 8%)	50 068 (94.5%)	<0.001^a^	26 045 (90.9%)	21 262 (92.7%)	<0.001^a^
No	2 649 (3.7%)	1 685 (3.2%)		1 725 (6%)	1 017 (4.4%)	
Unknown	1 760 (2.5%)	1 216 (2.3%)		894 (3.1%)	655 (2.9%)	
**Median Income** (2012–2016)**—n (%)**						
<$40.227	11 111 (15.8%)	8 506 (16.3%)	0.001^a^	4 393 (15.6%)	3 572 (15.8%)	0.85^a^
$40.227–$50.353	15 329 (21.8%)	11 686 (22.4%)		6 178 (21.9%)	4 939 (21.9%)	
$50.354–$63.332	16 994 (24.2%)	12 424 (23.8%)		6 833 (24.2%)	5 488 (24.3%)	
>$63.333	26 901 (38.2%)	19 552 (37.5%)		10 802 (38.3%)	8 584 (38%)	
**Location of Residence—n (%)**						
Urban	68 159 (98%)	50 448 (98.1%)	0.25^a^	27 374 (98.2%)	21 896 (98.1%)	0.51^a^
Rural	1 391 (2%)	980 (1.9%)		514 (1.8%)	430 (1.9%)	
**Charlson/Deyo Score—n (%)**						
0	50 647 (70.8%)	37 698 (71.2%)	<0.001^a^	23 470 (81.9%)	18 492 (80.6%)	<0.001^a^
1	12 472 (17.4%)	8 911 (16.8%)		3 407 (11.9%)	2 823 (12.3%)	
2	5 441 (7.6%)	4 251 (8%)		1 247 (4.4%)	1 150 (5%)	
>3	2 973 (4.2%)	2 109 (4%)		540 (1.9%)	469 (2%)	
**Year of diagnosis—n (%)**						
2004–2008	24 432 (34.2%)	18 189 (34.3%)	0.37^a^	10 933 (38.1%)	8 804 (38.4%)	0.62^a^
2009–2012	22 165 (31%)	16 517 (31.2%)		9 011 (31.4%)	7 118 (31%)	
2013–2016	24 936 (34.9%)	18 263 (34.5%)		8 720 (30.4%)	7 012 (30.6%)	
**Facility Type—n (%)**						
Cancer Program	37 636 (52.6%)	28 978 (54.7%)	<0.001^a^	9 242 (32.2%)	7 500 (32.7%)	0.15^a^
Academic/Research Program	29 895 (41.8%)	21 361 (40.3%)		9 340 (32.6%)	7 660 (33.4%)	
Unknown	4 002 (5.6%)	2 630 (5%)		10 008 (35.2%)	7 774 (33.9%)	
**Facility Region—n (%)**						
East North Central	11 274 (16.7%)	8 489 (16.9%)	0.006^a^	3 201 (17.2%)	2 631 (17.4%)	0.40^a^
East South Central	4 745 (7%)	3 682 (7%)		1 215 (6.5%)	1 065 (7%)	
Middle Atlantic	10 493 (15.5%)	7862 (15.6%)		2 727 (14.7%)	2 247 (14.8%)	
Mountain	3 351 (5%)	2 360 (4.7%)		961 (5.2%)	736 (4.9%)	
New England	3 718 (5.5%)	2 826 (5.6%)		860 (4.6%)	742 (4.9%)	
Pacific	8 305 (12.3%)	5 913 (11.7%)		2 222 (12%)	1 803 (11.9%)	
South Atlantic	14 144 (20.9%)	10 776 (21.4%)		3 865 (20.8%)	3 115 (20.5%)	
West North Central	5 415 (8%)	4 026 (8%)		1 990 (10.7%)	1 550 (10.2%)	
West South Central	6 086 (9%)	4 405 (8.8%)		1 531 (8.2%)	1 271 (8.4%)	
**Distance from Facility in Miles—Median (p25–p75)**	13.6 (5.7–34.1)	12.6 (5.3–32.5)	<0.001^b^	16.4 (6.5–43.8)	15.6 (6.4–41.7)	0.001^b^

^a^ Chi-square test.

^b^ Mann-Whitney Test (Wilcoxon Rank Sum).

The majority of GB patients received treatment (93% for males and 91.5% for females). Surgery was the most predominate treatment (76.3% for males and 73.7% for females), followed by radiation (71.1% for males and 68.1% for females), and then chemotherapy (65.3% for males and 61.3% for females) with a median time to treatment of 3 days for males and 2 days for females (**Table 2**). Differences between male and female GB patients existed for overall treatment receipt (p < 0.001), surgery (p < 0.001), time to surgery (p = 0.01), time to discharge (p < 0.001), unplanned 30-day readmission (p < 0.01), radiation (p < 0.001), days to radiation (p = 0.04), days of radiation (p < 0.001), chemotherapy (p < 0.001), vital status (p < 0.001), and 90-day mortality (p < 0.001). For LGG patients, the majority received treatment (92.4% for males and 92.2% for females) with the most predominate treatment being surgery (69.8 for males and 68.7% for females), followed by radiation (57.7% for males and 55.9% for females), and then chemotherapy (51.2% for males and 49.3% for females) with a median time to treatment of 6 days for both males and females. Differences for LGG patients existed for surgery (p = 0.04), time for discharge (p < 0.001), radiation (p < 0.001), chemotherapy (p < 0.001), vital status (p < 0.001), 30-day mortality (p = 0.003), and median survival (p < 0.001).

**Table 2:** Treatment characteristics by sex for GBM and LGG, National Cancer Database (NCDB), 2004–2016.

	Glioblastoma			Lower Grade Glioma		
	Male	Female	P value	Male	Female	P value
**Treatment—n (%)**						
Yes	38 873 (93%)	28 186 (91.5%)	<0.001^a^	14 270 (92.4%)	11 389 (92.2%)	0.49^a^
No	2 510 (6%)	2 316 (7.5%)		834 (5.4%)	673 (5.4%)	
Active Surveillance	61 (0.1%)	57 (0.2%)		194 (1.3%)	168 (1.4%)	
Unknown	344 (0.8%)	256 (0.8%)		137 (0.9%)	129 (1%)	
**Days from diagnosis to treatment—median (p25–p75)**	3 (0–10)	2 (0–10)	0.08^b^	6 (0–28)	6 (0–29)	0.32^b^
Standard of care treatment* for GBM only—n (%)						
Yes	36 741 (51.8%)	25 334 (48.2%)	<0.001^a^	–	–	–
No	34 192 (48.2%)	27 175 (51.8%)		–	–	–
**Surgery—n (%)**						
Total Resection	13 361 (18.7%)	9 619 (18.2%)	<0.001^a^	4 306 (15%)	3 518 (15.3%)	0.04^a^
Partial Resection	12 067 (16.9%)	8 435 (15.9%)		3 983 (13.9%)	3 068 (13.4%)	
Local Excision	6 379 (8.9%)	4 691 (8.9%)		2 538 (8.9%)	2 010 (8.8%)	
Other Surgery	22 742 (31.8%)	16 245 (30.7%)		9 171 (32%)	7 155 (31.2%)	
No	16 897 (23.6%)	13 930 (26.3%)		8 592 (30%)	7 116 (31%)	
Unknown	87 (0.1%)	49 (0.1%)		74 (0.3%)	67 (0.3%)	
**Days from diagnosis to surgery—median (p25–p75)**	1 (0–6)	1 (0–6)	0.01^b^	2 (0–17)	2 (0–18)	0.40^b^
**Days until discharge—median (p25–p75)**	4 (2–6)	4 (3–6)	<0.001^b^	3 (2–5)	3 (2–5)	<0.001^b^
**Unplanned readmission after 30 days—n (%)**						
Yes	2 686 (3.8%)	1 970 (3.7%)	0.01^a^	802 (2.8%)	610 (2.7%)	0.16^a^
No	66 479 (92.9%)	49 398 (93.3%)		27 042 (94.3%)	21 619 (94.3%)	
Unknown	2 368 (3.3%)	1 601 (3%)		820 (2.9%)	706 (3.1%)	
**Radiation—n (%)**						
Yes	50 857 (71.1%)	36 051 (68.1%)	<0.001^a^	16 553 (57.7%)	12 827 (55.9%)	<0.001^a^
No	20 267 (28.3%)	16 628 (31.4%)		11 794 (41.1%)	9 847 (42.9%)	
Unknown	409 (0.6%)	290 (0.5%)		317 (1.1%)	260 (1.1%)	
**Days from diagnosis to radiation—median (p25–p75)**	32 (24–42)	32 (23–42)	0.04^b^	40 (28–61)	40 (28–61)	0.44^b^
**Days of radiation—median (p25–p75)**	43 (40–46)	43 (39–46)	<0.001^b^	44 (41–47)	44 (40–47)	0.09^b^
**Chemotherapy—n (%)**						
Yes	46 697 (65.3%)	32 492 (61.3%)	<0.001^a^	14 663 (51.2%)	11 297 (49.3%)	<0.001^a^
No	22 457 (31.4%)	18 637 (35.2%)		12 749 (44.5%)	10 621 (46.3%)	
Unknown	2 379 (3.3%)	1 840 (3.5%)		1 252 (4.4%)	1 016 (4.4%)	
**Days from diagnosis to chemotherapy—median (p25–p75)**	31 (21–43)	31 (21–42)	0.81^b^	40 (26–68)	40 (26–69)	0.52^b^

*Standard of care treatment is equal to Stupp protocol (Chemotherapy + Surgery + Radiotherapy).

a Chi-Square Test.

b Mann-Whitney Test (Wilcoxon Rank Sum).

**Figure 1:**
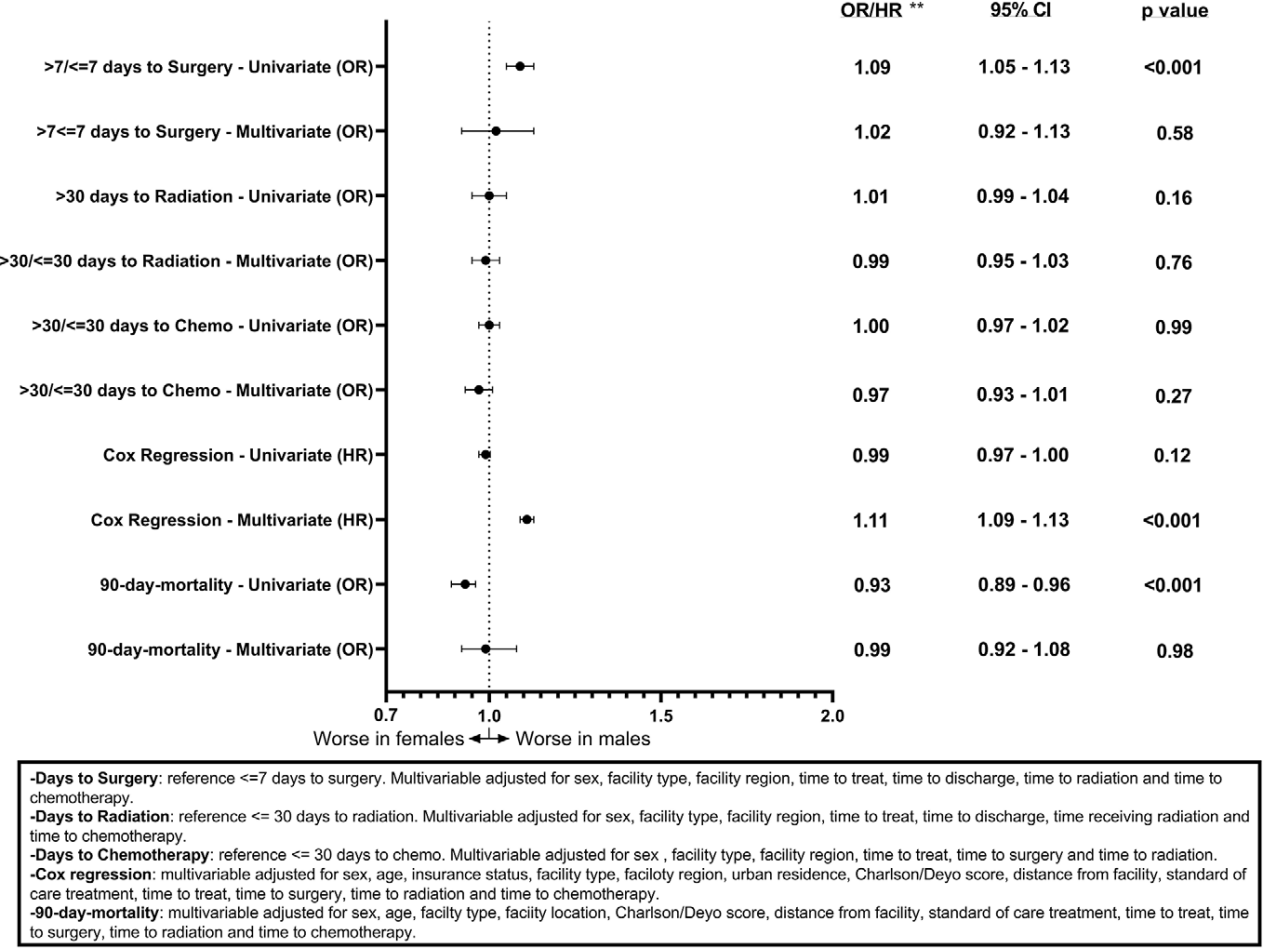
Forest plot of sex differences in clinical outcomes for GBM, National Cancer Database (NCDB), 2004–2016. **Odds ratios from logistic regression for days to surgery, days to radiation, days to chemotherapy, and 90-day mortality and Hazard ratios from Cox regression for overall survival.

**Figure 2:**
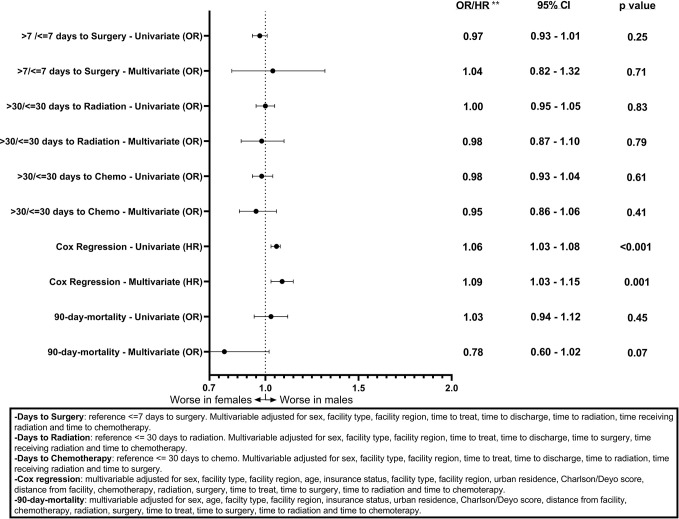
Forest plot of sex differences in clinical outcomes for LGG, National Cancer Database (NCDB), 2004–2016. **Odds ratios from logistic regression for days to surgery, days to radiation, days to chemotherapy, and 90-day mortality and Hazard ratios from Cox regression for overall survival.

Associations between sex and days to surgery, days to radiation, and days to chemotherapy for GB patients are shown (**Figure 1**). Males had a statistically significant association only with >7 days to surgery (OR = 1.09, CI 1.05–1.13, p < 0.001) on the univariable model but this association was no longer present when using the multivariable model (OR = 1.02, CI 0.92–1.13, p = 0.58). **Figure 2** shows the associations between sex and days to surgery, days to radiation, and days to chemotherapy for LGG. No significant associations were encountered.

**Figures 1** and **2** also show sex differences in overall survival and 90-day mortality for GB (**Figure 1**) and LGG (**Figure 2**). For GB patients the median survival was 9.72 months for males compared to 8.67 months for females (p = 0.12). The median survival for LGG patients was 48.66 months for males *versus* 55.26 months for females (p < 0.001) (**Table 3**). After adjustment for key variables including time to treatment variables, multivariable Cox proportional hazards regression models for overall survival showed that GB males had a higher risk of death (HR = 1.11, CI 1.09–1.13, p < 0.001) as did LGG males (HR = 1.09, CI 1.03–1.15, p < 0.001). When assessing sex differences in 90-day mortality, there were differing results for GB compared to LGG when using the univariable models but remained the same when using a multivariable model. For GB, the univariable logistic regression model showed sex differences in 90-day mortality (OR for males = 0.93, CI 0.89–0.96, p < 0.001) but these sex differences where no longer present after adjustment (OR for males = 0.99, CI 0.92–1.08, p = 0.98). There were no observable sex based differences in either the univariable or multivariable logistic regression models in 90-day mortality for LGG patients (OR for males = 1.03, CI 0.94–1.12, p = 0.45; multivariable OR for males = 0.78, CI 0.60–1.02, p = 0.07).

**Table 3:** Survival characteristics by sex for GBM and LGG, National Cancer Database (NCDB), 2004–2016.

	Glioblastoma			Lower Grade Glioma		
	Male	Female	P value	Male	Female	P value
**Vital Status—n (%)**						
Alive	6 061 (9.3%)	4 892 (10.1%)	<0.001^a^	12 481 (46.9%)	10 461 (49.1%)	<0.001^a^
Dead	58 995 (90.7%)	43 418 (89.9%)		14 161 (53.1%)	10 865 (50.9%)	
**Thirty Day Mortality—n (%)**						
Yes	2 557 (5.1%)	1 778 (5%)	0.63^a^	421 (2.3%)	263 (1.8%)	0.003^a^
No	46 824 (94.9%)	33 560 (95%)		18 093 (97.7%)	14 273 (98.2%)	
**Ninety Day Mortality—n (%)**						
Yes	7 425 (15%)	5 677 (16%)	<0.001^a^	942 (5%)	727 (5%)	0.74^a^
No	41 956 (85%)	29 661 (84%)		17 572 (95%)	13 809 (95%)	
**Median Survival—months (95% CI)**	9.72 (9.59–9.82)	8.67 (8.51–8.80)	0.12^b^	48.66 (46.88–50.23)	55.26 (52.83–57.92)	<0.001^b^

a Chi-Square Test.

b Log-Rank Test.

## Discussion

The primary objective of this work was to assess sex differences in time to treatment and assess the potential effects of these differences on survival for GB and LGG. The selection criteria led to the use of a large and robust population (176,100 patients) from the NCDB, with a predominance of GBM diagnoses in the period (70% of the study population), corroborating the CBTRUS epidemiology, which classifies it as the most common glioma (1).

This study showed that, like other cancers, GB and LGG occurs more in males than in females (17). The biological mechanisms to explain these sex differences of GB and LGG are not fully understood. One study showed that this sex difference may be partially explained through increased malignant transformation susceptibly in male astrocytes with loss of p53 function when compared to female astrocytes with a loss of p53 function (18). Research is focused on the involvement of other genes, which likely act in conjunction with hormonal and environmental factors to explain the observed sex differences in occurrence (17).

Our study showed that males receive, on average, more treatment than females, with higher rates of radiotherapy, chemotherapy, and surgery for both GB and LGG patients. In a previous study using NCDB data, GB patients who received treatment from an academic facility had a higher likelihood of undergoing surgery and adjuvant therapies (19, 20). The multivariable analysis of days to treatment, adjusting for confounding variables, shows that males with GB are associated with a greater chance of >7 days to surgery. Such findings show that, although more men receive treatment, their treatment may start later, which may contribute to the differences in survival between the sexes. One factor that may explain this longer time might be the differences in distance from treatment facility and insurance coverage between the sexes. Survival analysis showed that for both GB and LG, males have a higher mortality hazard ratio in multivariate analyses, corroborating previous studies that found female superiority in terms of survival (4), thus adding evidence with a larger population. Sex was not significant on univariate survival analysis, however there were significant survival differences by sex on multivariate model reflecting the true effect of sex. Our study uses a large database of patients and corroborates previous smaller studies that demonstrate more males have GB and LGG in males while females have better survival. We add to our analysis an important factor that could be associated with outcomes, multiple time to treatment variables. Based on our results, we believe that differences in treatment and/or treatment time between the sexes, while statistically significant, are small and insufficient to fully explain the differences in outcomes by sex.

This study has several limitations. Our database (NCDB) is based on 1,500 US hospitals, and while it captures 70% of cancer cases in the US there are certain regions of the country that are under or overrepresented due to variable hospital network participation, thus our selection does not indicate a population sample outside this context. Unfortunately, due to very low incidence of these tumors, we had very few patients for majority of hospitals included in the analysis. With a larger sample size per hospital, a mixed model approach is superior to account for within hospital variation. However, we included facility type and facility region to control for it. In addition, we did analyze patients according to mutations, health status, or degree of cancer progression at the time of diagnosis, and these may be confounding factors that alter outcomes and the most urgent need for treatment. Details for the types of treatment administered are not specified, such as specific chemotherapeutic agents per patient, and it is possible that these specific details may impact sex differences in outcomes. Additional studies with other databases are needed to analyze factors not considered here in order to provide more information on sex differences in treatment time and outcomes for gliomas.

In summary, we found that males have a higher proportion of GB and LGG compared to females, in addition to receiving, on average, higher rates of radiotherapy, chemotherapy and surgery, yet having a higher risk of death. In addition, we demonstrate that there are no statistically significant differences in multiple time to treatment related variables when comparing by sex.

## Data Availability Statement

Publicly available data sets were analyzed in this study. This data can be found here: https://www.facs.org/quality-programs/cancer/news/ncdb-puf-090220.

## Author Contributions

NS: data management, statistical analysis, paper writing. HK: paper writing. NP: data management. KW: paper writing. JB-S: data management, statistical analysis, paper writing. All authors contributed to the article and approved the submitted version.

## Conflict of Interest

The authors declare that the research was conducted in the absence of any commercial or financial relationships that could be construed as a potential conflict of interest.
